# Evaluation of Cleaning Processes Using Colorimetric and Spectral Data for the Removal of Layers of Limewash from Medieval Plasterwork

**DOI:** 10.3390/s20247147

**Published:** 2020-12-13

**Authors:** Miguel Ángel Martínez-Domingo, Ana Isabel Calero Castillo, Eva Vivar García, Eva M. Valero

**Affiliations:** 1 Department of Optics, Faculty of Sciences, University of Granada, 18071 Granada, Spain; valerob@ugr.es; 2 Department of Painting, Faculty of Fine Arts, University of Granada, 18071 Granada, Spain; anacalero@ugr.es (A.I.C.C.); evavivar@correo.ugr.es (E.V.G.)

**Keywords:** spectral imaging, plasterwork, restoration, cleaning procedures, lime layer

## Abstract

In the cultural heritage preservation of medieval buildings, it is common to find plaster walls covered in lime, which previously were painted in polychromy. The conservation interventions usually try to remove the whitewash, whilst maintaining the original color of the painted wall as much as possible. However, there is no agreement on which cleaning technique best preserves the original appearance of the colored plaster. Different pigments found below the lime layer may behave differently depending on the cleaning technique used. Usually, colorimetric or photometric area-based measurements are carried out to study the color of the cleaned areas to compare with their original color, obtained from pre-made plaster probes. However, this methodology fails when the mean color difference is not enough to fully characterize the changes in texture and color appearance. This study presents a set of experiments carried out using two different pigments (cinnabar and malachite) covered with lime, and treated with nine different cleaning techniques on plaster probes prepared according to medieval techniques. We have studied the effect of the cleaning process on the color and the homogeneity of the samples using a hyperspectral imaging workflow. Four different analysis methods are presented and discussed. Our results show that the proposed analysis is able to provide a much more comprehensive and diversified characterization of the quality of the cleaning method compared to the commonly used colorimetric or photometric area-based measurements.

## 1. Introduction

**Figure 1:**
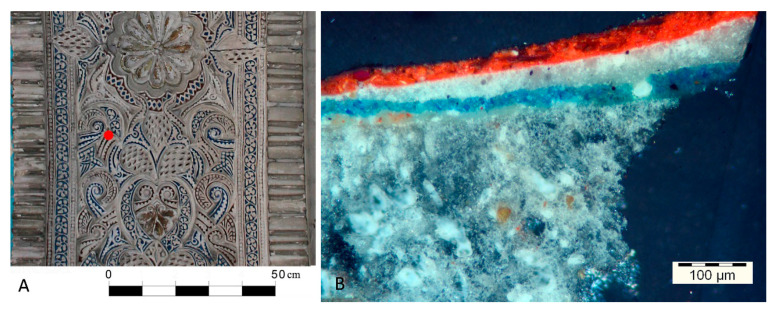
(**A**) Plasterworks in the intrados of the access gate of Carlos V room in Real Alcázar of Sevilla. The red dot highlights the area where the sample was collected. (**B**) Stereo microscope image of a sample taken from the Patio de las Doncellas in the Real Alcázar in Sevilla where there is presence of whitewash and red polychromy hiding its original blue color.

One of the main problems that medieval plasterwork presents, as far as its conservation and restoration is concerned, is the risk involved with the cleaning processes used. Currently, the great majority of plasterwork decorations show a very different aspect to what they originally had, which was characterized by vivid, rich colors similar to those found today in ceramic tiles [1]. Historically, layers of various materials were applied one on top of the other to change the original appearance of the plasterwork. On the one hand, they were whitewashed to adapt to the neoclassical taste or for hygiene reasons, or on the other hand, the polychromy was redone to refresh or renovate colors which had been lost over time. An example of this kind of actions from the plasterworks of patio de las Doncellas in Real Alcázar of Sevilla is shown in (Figure 1) [2].

In these cases, from a conservative point of view, we consider the alteration a dirt problem as it has to be removed with a cleaning process. This is where we can encounter great difficulties because when we remove the altered layers the original polychromy can be detached too, and it is therefore very difficult to remove them without negatively affecting the original piece [3]. Due to the current complexity of their removal and the extent of the topic, this study focuses only on cleaning the whitewash layers. In this sense, it is important to highlight that it is a topic which has not been extensively addressed, and there are only few studies which have focused on this problem affecting this type of decoration.

The difficulties encountered removing this kind of alteration have been analyzed in previous studies, such as the ones conducted by Hubbard [4] or Cotrim et al. [5], among others. The problem of removing this kind of modifications is the need of using solvents that are invasive for the plasterwork as well. This is the case of deionized water and other polar solvents such as acetone or alcohols like ethanol [4,5,6]. In order to avoid the excessive pervasion of these methods, cotton poultices were used traditionally [4], or more recent techniques such as gels [5]. In these works, the authors highlight the need of combining those chemical treatments with the use of mechanical procedures, such as the scalpel, for the cleaning to be effective, especially in case of gels [5].

Some of the most relevant interventions dealing with the issue of whitewash removal are the plasterwork restoration in the Madraza chapel [2], the plasterwork restoration in the hall of the Palace of King Don Pedro in Seville [7] and the intervention in the gothic linear style murals in the Santa Maria la Nueva church in Zamora [8], or the restoration of the plasterwork front in a Nasrid building of XIV–XV centuries in Granada [9]. These works highlight the problem of the existing limewash layers and the difficulty of their removal. On the one hand, the limewash layer causes the decohesion of the inner layers agglutinated with organic materials (animal glue, Arabic gum, and egg). Thus, during the removal process, there is the risk of removing the original layer together with the limewash layer. On the other hand, it is also highlighted in these works that the presence of such layers, involves a problem of the chronological decontextualization of the coating. This makes it necessary to remove them, in order to preserve and date the pieces. The cleaning methods applied on these works, are based on both the use of manual cleaning methods with scalpels [2,7], and the use of these techniques combined with chemical treatments such as applying solvents like water and ethanol with cotton poulettes [8,9]. In those cases where only mechanical procedures were applied, it was concluded that they are very aggressive and they may remove part of the original polychromy layer as well. Hence, the need of including chemical treatments which make the cleaning more effective and decrease the risk of introducing changes in the original color [2,7]. Accordingly, in those works where a mechanical and chemical combination of procedures were applied, the cleaning was much more effective and the appearance of the original color was better preserved [8,9].

The aim of this study is to determine the effectiveness of a series of cleaning processes that allow the removal of whitewash layers from polychromed plasterwork. The effectivity of the cleaning methods over polychromic layers has been evaluated in other studies through different techniques like stereo microscopy, optical microscopy, scanning electron microscopy, photogrammetry, or colorimetric point measurements using a spectrophotometer [10,11,12]. While the colorimetric point-based measurements only offer information about the mean changes produced in a specific area of the sample, imaging techniques like the ones presented in this work provide more detailed information about the local changes produced in the surface.

To conduct the research, we used plaster probes which underwent a 36-month ageing process and simulated the materials and execution techniques of medieval plasterwork. Then, we added a layer of whitewash and selected various cleaning processes in order to test them. The base materials were selected taking into account previous work conducted by our team and other researchers in some of the most representative monuments of this period: Cuarto Real de Santo Domingo [13], the Madraza chapel [2], the plasterwork of the Alhambra [14,15], and the Real Alcazar of Sevilla [16]. Regarding the cleaning processes, we selected traditional methods, both physical (such as a scalpel) and chemical, and other techniques more recently used in restoration (polysaccharide-based gels, polyacrylic acids, or cellulose ethers).

We should highlight the complexity of assessing colorimetric data objectively in these kinds of studies. The simple visual analysis of the samples or the analysis of images taken with a camera, although useful, do not offer an objective assessment in this kind of work, where the color differences between two samples measured with the tools traditionally used for this aim (point/area-based measurements) can be minimum, although both samples are visibly very different.

The aim of this work is to assess the effectiveness of these cleaning processes in very precise areas of the probes. For such purpose, we propose a method based on the use of perforated acetate templates and hyperspectral imaging techniques to analyze in more detail the changes produced in the studied chromatic surfaces.

Analysis methods based on spectral images have been successfully applied in previous studies for restoration and cultural heritage applications. For example, in [17], the effect of different consolidants used in medieval plasterwork is analyzed. In [18], the effect of ageing of the different varnish materials is studied on paper samples. In [19], a complete capturing and processing workflow is presented for the high dynamic range and multiple focus hyperspectral imaging of works of art. However, to the best of our knowledge spectral imaging has not so far been used to assess the quality of cleaning procedures in plaster probes. In this study, four novel analysis techniques based on spectral imaging data are proposed to study the performance of nine different medieval plasterwork cleaning methods aimed at restoring the original appearance of the samples. Most of the methods presented here would require the use of either calibrated imaging or spectral capture devices. This makes spectral imaging devices a very convenient tool for analyzing the effect of the cleaning procedures on our samples, since spectral imaging techniques allow us to perform conventional simple colorimetric analyses based on point color measurements and also to have access to colorimetric pixel-by-pixel information.

The remainder of the paper is organized as follows: in Section 2 we describe the experimental methods used, including the sample preparation and spectral imaging capture devices used. In Section 3, we present the main results of the different analytical methods proposed. In the discussion section (Section 4) we extend the analysis of the results presented, and finally in the conclusions section (Section 5) we summarize the main outcomes of this work.

## 2. Materials and Methods

### 2.1. Plaster Probes

For this study two probes reproducing medieval plasterwork were used. The probes were chosen due to the necessity of having a wide colored surface on which to be able to assess objectively and precisely the different processes to be tested. To make the probes the results from 10 samples analyzed with X-ray diffraction from the plasterwork of the Patio de las Doncellas in the Real Alcázar de Sevilla [16] were taken as a reference. The probes were made using a base of fired clay bricks manufactured industrially, over which the plaster support was reproduced with white gypsum plaster 95% (CaSO_4_•½H_2_O) certified by AENOR, to which 5% calcium hydroxide (CaCO_3_), was added (from CALCINOR). The amounts used were 500 g of calcium sulphate, 25 g of calcium hydroxide and 1200 mL of water using a mold in order to be able to repeat the probes, which were 20.5 cm long, 10 cm wide, and 2.7 cm thick.

**Figure 2:**
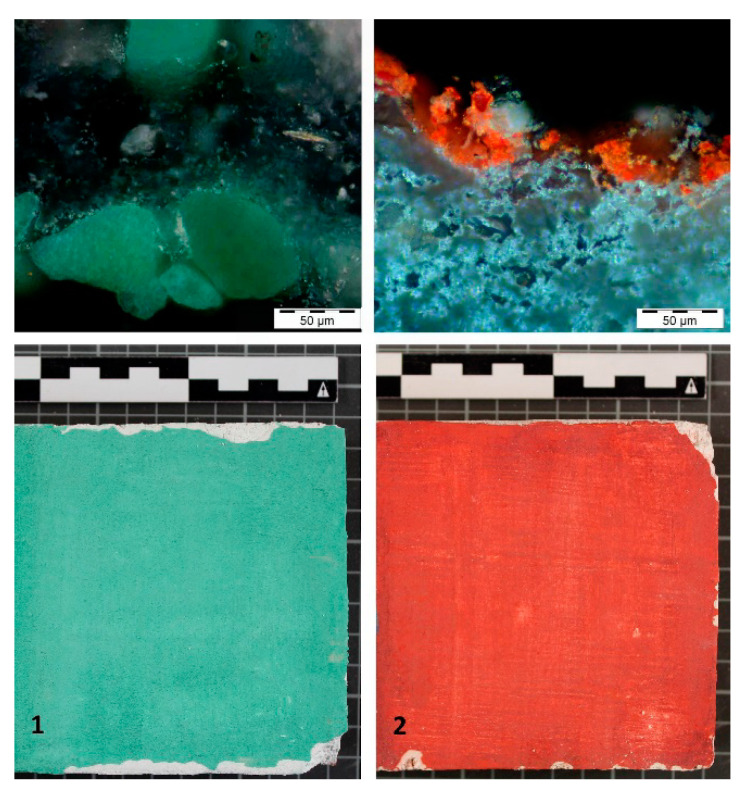
Above: identification of natural malachite and cinnabar in the original layers of the plasterwork in the Patio de las Doncellas in the Real Alcázar of Seville. Below: general image of the chromatic study surface of the probes.

On this support a layer of polychromy is added, based on the tempera technique, which uses a pigment and a binder. The used pigments are natural malachite (K.10300) for probe 1 and cinnabar (K.42000) for probe 2 from Kremer Pigmente GmbH & Co.KG^®^ (Aichstetten, Germany) manufacturers. These pigments were selected taking into account the results from previous studies, as they are two of the most commonly found in work of this period [2,13,14,15,16]. Two binders were selected. On the one hand, animal glue for probe 1 and gum arabic for probe 2, as they were the most used during this period. Both are from manufacturers CTS^®^ (Madrid, Spain) and were prepared at 10% concentration (Figure 2).

The proportion of pigment and binders was determined with the aim of creating a homogenous, opaque and covering layer over the plaster support to be able to assess adequately the data obtained from the cleaning tests. The proportion for the natural malachite was 10 mL/5 g, whilst the proportion of the cinnabar pigment was 10 mL/3 g [16].

The prepared chromatic surfaces were aged for 36 months after their preparation with polychromy. After applying the whitewash, they were aged for 3 months before proceeding with the cleaning processes. The aging process consisted in storing the probes in a research laboratory, controlling the humidity and temperature (daily, weekly, and monthly) using a portable device by Sensonet. This procedure allowed us to control the real conditions of the probes during aging, ensuring the effectivity of this kind of natural aging method (EEA) for the evaluation of polychromic surfaces, which was used in previous studies [20].

### 2.2. Acetate Template Evaluation

**Figure 3:**
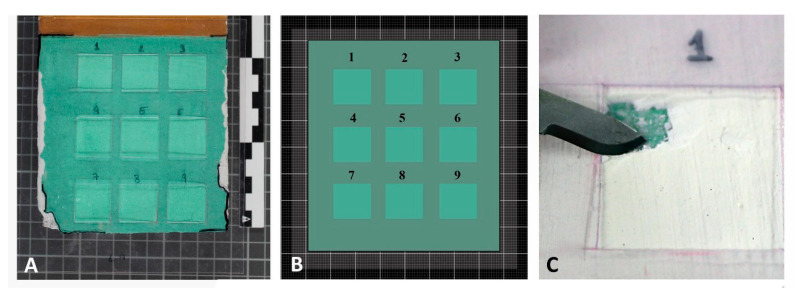
(**A**) Image of Probe 1 in its initial stage with the acetate template that shows the gaps where the different cleaning processes will be applied. (**B**) Two-dimensional and simplified simulation of Probe 1 and how the gaps of the acetate template created for the cleaning tests are distributed. (**C**) Mechanical cleaning process with a scalpel.

Due to the complexity of the cleaning processes it was essential to define exactly the areas of polychromy where the different treatments were applied to be able to extract the relevant data. So, following the methodology recommended by the Institute of Spanish Cultural Heritage in the project Nanorestart—EU Project (Nanomaterials for the Restoration of Works of Art) [21]—for interventions in contemporary works of art, we decided to design acetate transparent templates adapted to the surface of each assessed probe. In one of these templates 9 holes were made and each was assigned a selected cleaning process with a test area of 2 cm^2^. In each of these the number of the process was indicated with a permanent marker so that the condition before and after the treatment could be perfectly compared in each of the chromatic test surfaces (Figure 3).

### 2.3. Cleaning Tests

As we mentioned before, the fact that there are few studies focusing on adequate cleaning processes to remove whitewash from medieval polychromed plasterwork was one of the biggest problems of this study. Therefore, it was necessary to do an in-depth review of the existing bibliography on cleaning treatments that dealt with the problem of removing a superficial layer when the base material is plaster, soluble in water, painted with the tempera technique (with gum arabic or animal glue) with water-soluble binders. After this review two types of techniques were chosen—mechanical and chemical—which were applied individually or in combination (Table 1).

To date, physical treatments have been the most used to remove both whitewash and calcium carbonate in archaeological mural paintings. However, they are very abrasive methods that depend greatly on the restorers’ expertise, and are also not advisable when the surface is very wide because of the time it takes. Among these, the most used are mechanical/manual techniques such as surgical knifes, scalpels, glass fiber pencils, or wood sticks among others; or electronic tools such as small drills which are faster than the first but are sometimes not advisable when removing thin layers on weak polychromy [22]. Gradually, new techniques such as the infrared laser have been used recently. This gives good results in some cases, but it is expensive, which can be a problem particularly in small restoration interventions [23,24].

For this study, we chose the scalpel and the glass fiber pencil from the mechanical techniques described above due to the advantages both methods have for removing layers of this type from wall coverings [25]. Both were tested individually: (method 1) scalpel and (method 9) glass fiber pencil or combined with other methods as described in Table 1.

We will now go on to describe the chemical methods that were used. The use of solvents such as deionized water, triammonium citrate, ethanol, acetone, toluene, or white spirit, have been traditionally used for these decorations to soften the surface which has to be removed. The revised bibliography recommends using these solvents with a thickening or gelling agent to improve control and effectiveness. Traditionally, cellulose or cotton poultices have been used and recently gels made with polyacrylic acids, complex polysaccharides, and cellulose ethers have been incorporated [6,26,27,28].

For this study, from the above methods we chose distilled water for method 2 (Probe 1) and a 75/25 mixture of water and acetone (Probe 2), taking into account the positive effects of these solvents which have been highlighted by authors such as Hubbard [4], Wolbers [29], Bogiorli [28], Giordano and Cremonesi [30] or Tortajada and Blanco [31]. To apply them a selection of the most adequate gelling and thickening agents for surfaces sensitive to water, such as plaster, was carried out. These were applied in both probes (Probe 1 and Probe 2) in the different cleaning processes that were used: cellulose poultice (method 3), gel formed from the polyacrylic acid Carbogel^®^ (CTS^®^, Madrid, Spain) (method 4), gel formed from the complex polysaccharide Gellano Kelogel^®^ (CTS^®^, Madrid, Spain) (method 5), cotton swab (method 6), gel formed from the complex polysaccharide Agar-Agar (CTS^®^, Madrid, Spain) (method 7), and gel formed from the hydroxypropyl cellulose Klucel G^®^ (CTS^®^, Madrid, Spain) (method 8). With the aim of widening the study and observing the effects of different processes, we decided to change the mechanical process based on a scalpel applied in method 1 for Probe 1, for another more innovative process based on the use of another thickening agent, specifically a cellulose ether, as it has not been studied in medieval plasterwork to date. The agent chosen to form a gel was the methylcellulose Culminac MC2000^®^ (CTS^®^, Madrid, Spain) used in method 1 for Probe 2.

On the other hand, within the chemical methods, the use of acids and bases should also be highlighted to remove carbonate crusts as they have been frequently used for this kind of interventions. However, their use should be limited as they are very aggressive on surfaces with delicate polychromy [32]. Within this group, ethylenediaminetetraacetic acid (EDTA) is often used to remove whitewash and carbonate crusts [12]. Its use, as one of the main components of the AB-57 poultice (I.C.R. formulation-Rome), created by the Mora brothers [33], has widely demonstrated its effectiveness removing this kind of alterations on wall coverings as it hydrolyses the fats that are present between the painted surface and the carbonate crusts, allowing the removal of the latter. Taking this into account, checking the effectiveness of the AB-57 (30 g of ammonium carbonate, 50 g of sodium bicarbonate, 25–100 g of tetrasodium EDTA (pH 11), 25 g of the surfactant New Des 50^®^ at 10%, 6 g of carboxymethylcellulose) was essential for this study (method 3).

In all the tests, except the mechanical ones, Japanese paper or tissue was inserted between the artwork and the material to protect the surface, avoid direct contact and improve the removal [22]. As indicated in Table 1, in all the mentioned tests we decided to combine the chemical cleaning treatments with a subsequent mechanical treatment using a scalpel due to the good results obtained with both techniques. Examples of this are the intervention conducted on the plasterwork of the facade of the Alcazar of Seville [34] and on the plasterwork of the cloister of the Cathedral of Toledo [35].

### 2.4. The Colorimetric and Spectral Analysis Method

Hyperspectral images of the probes were captured before and after the cleaning process using a hyperspectral imaging scanner model Resonon Pika L. This scanner yields hyperspectral images roughly in the range from 383 to 1016 nm, with 4.1 nm spectral resolution. The system is calibrated for illumination and flat field correction, so the hyperspectral cubes contain pixel-wise spectral reflectance information of the probes [17,18,36].

**Figure 4:**
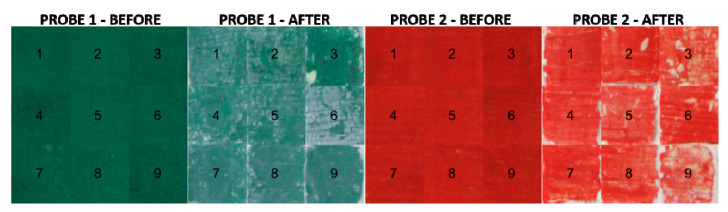
sRGB renderization of the 100 × 100 pixel areas extracted from all the samples both before and after the cleaning process.

For each of the 9 samples in each probe, an area of 100 × 100 pixels was extracted both before and after the cleaning process. The sRGB renderization [37] of these areas is shown in Figure 4.

Once the areas are extracted, their spectral reflectances are interpolated to the range from 400 to 720 nm in 1 nm step. In this way we are able to retrieve the reflectance information only in the visible range. The mean spectral reflectances as well as the wavelength-wise standard deviations are then calculated for the selected 10,000 (100 × 100) pixels in each area. The CIE XYZ tristimulus values and the CIE L*, a*, b* color coordinates are also calculated pixelwise under CIE D65 standard illuminant and using a reference white tile from Sphere Optics. Using the CIE L*, a*, b* data, we can calculate both the volumes of the L*, a*, b* clouds, as well as the mean color of each sample (a point-measurement device such as a spectrophotometer would do). The former is calculated using Delaunay’s triangulation method [38], with infinite radius, together with an auxiliary algorithm based on the alpha-shape concept [39].

For all the samples, 2 spectral metrics (Goodness of Fit Coefficient (GFC) and Root Mean Square Error (RMSE)) [40] and one color metric (CIEDE 2000 color difference) [41] were calculated to compare the reflectance spectra as well as the color of the samples both before and after the cleaning process.

Finally, a k-means algorithm [42] was performed over the L*, a*, b* data clouds to automatically segment those areas of residual white present in the samples after the cleaning process. These areas are present due to two different reasons: firstly, some residue of the top white layer may remain even after the cleaning process; and secondly the cleaning process could be so aggressive that even the pigment was eliminated, and therefore the underlying white plaster material becomes visible. In both cases we would consider white areas to be undesirable. It is then important to be able to quantify the portion of the sample that remains white after the cleaning procedure, and this can be done by calculating the number of pixels that are classified as white using the k-means algorithm.

To evaluate the performance of the k-means classification, a set of manually selected pixels were extracted from the samples as a ground truth. These pixels consisted of areas of the samples clearly containing white areas or pigment areas. K-means was applied to this ground truth set to check its performance.

## 3. Experimental Data and Results

### 3.1. Mean Spectral Reflectance, Spectral Standard Deviation, and Spectral Metrics Results

**Figure 5:**
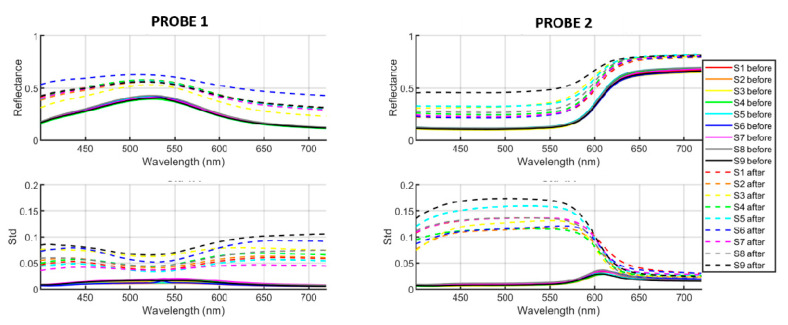
Mean spectral reflectances (top row) and wavelength-wise standard deviations (bottom row) of the 36 samples shown in Figure 4. Left column for probe 1 and right column for probe 2. Continuous lines represent the samples before and dashed lines after the cleaning process.

The mean spectral reflectances and the wavelength-wise standard deviation of the 36 samples (see Figure 4) are shown in Figure 5. Continuous lines represent the samples before and dashed lines after the cleaning process.

Note how all samples of the same probe have almost the same reflectance before cleaning, with very low standard deviation for all the wavelengths, and how after cleaning the mean reflectances get lighter (due to the whiter regions) and more spread across the samples. The standard deviation also gets much higher for each sample due to the heterogeneity introduced by the cleaning processes. The cleaning process that results in the highest standard deviation is number 9 (glass fiber pencil) for both probes 1 and 2. The standard deviation is clearly dependent on wavelength for both samples. The wavelength ranges for which the sample has a lower reflectance also register a higher standard deviation (blue/red spectral ranges for the green sample, blue/green spectral ranges for the red sample). This can be explained if we consider that after cleaning the spectral reflectance tends to be flatter (approaching the typical shape of a white color). The initial spectral reflectance of the samples is closer to the flat shape representative of the white for those wavelengths which have a higher signal, and so to approach the flat curve, the variation introduced in the spectral ranges for which the signal is lower needs to be higher, and thus the standard deviation is also higher in the low signal range.

Regarding the effect of lightness increase, procedure number 6 (cotton swab + scalpel) results in the highest increase for probe 1 and procedure number 9 (glass fiber pencil) yields the highest lightness for probe 2. This can be explained if we consider that the increase in lightness is not always due to the appearance of white areas, but it can also be produced by a more uniform but imperfect cleaning of the sample, which would not necessarily increment the standard deviation across the pixels.

We have as well performed an additional analysis using the spectral reflectance information on a pixel-by-pixel basis and two relevant spectral metrics: GFC and RMSE. We have compared the pixel-by-pixel reflectance spectra of each Probe after the cleaning with the mean spectral reflectance spectrum of each original Probe (before the limewash covering and cleaning). For Probe 1, the method of choice would be number 3 (mean RMSE of 0.193, mean GFC of 0.9928), and the worst method number 6 (mean RMSE of 0.372 and mean GFC of 0.9671). Both metrics are in agreement for Probe 1. For Probe 2, the best method would be number 2 followed closely by number 1, 6, and 7 according to RMSE results (mean RMSE of 0.218). GFC metric results are better for method number 6 (with average GFC of 0.9856), while the worst method would be number 9 according to both metrics (with average RMSE of 0.406 and average GFC of 0.9198). The cleaning procedures have introduced changes both in scale and shape of the spectral reflectances, but the changes in shape are in general more relevant, especially for Probe 2.

### 3.2. Mean L*, a*, b* Values

We analyze now the L*, a*, b* mean values of the samples before and after the cleaning procedures. In Table 2, the mean CIEDE00 color difference introduced by the cleaning procedures with respect to the original samples is shown.

The information shown in Table 2 is what a standard point-measurement device such as a colorimeter or spectrophotometer able to measure the full area of the sample would deliver. We see how for probe 1, the best mean-color-preserving cleaning method is number 3 (AB-57 + scalpel), and the worst is number 6 (cotton swab + scalpel, also producing the highest increase in mean spectral reflectance), whilst for probe 2, the best performing methods in terms of mean color difference are methods 1 (methylcellulose gel + scalpel), 2 (cellulose poultice + scalpel), and 7 (complex polysaccharide gel (Agar-Agar^®^) + scalpel), and the worst is method number 9 (glass fiber pencil). Comparing two by two all the nine samples before cleaning, the mean color difference was 0.9 units, with a standard deviation of 0.49 for probe 1, and 1.2 units with a standard deviation of 0.63 for probe 2. This points to the fact that probe 2 was more heterogeneous before the cleaning process, and also that in general, probe 2 has been more affected by the impact of the cleaning processes. All the color differences obtained after the cleaning procedures are clearly above the usual threshold for CIEDE2000 data, meaning that the color difference between the sample both before and after cleaning would be visually perceptible. 

Table 3 shows the differences in average color coordinates L*, a* and b* for the 2 probes and the 9 cleaning methods.

As Table 3 shows, for Probe 1, the method of choice and the worst method would be the same as those selected considering only the color difference data shown in Table 2. For Probe 2, the worst method would be still number 9, but the best would be number 6. However, when looking only at the color differences shown in Table 2, there were three possible methods of choice (1, 2, and 7) with the same color difference. According to the differences in mean a* and b* values, the method of choice for Probe 2 would be number 6, which was not selected according to the mean color difference results, although it offers comparative results to the ones chosen for this Probe. The results in Table 3 allow us to characterize as well the change in color towards less saturated colors after the cleaning. Specifically, for Probe 2 all Δa* and Δb* values are negative, and for Probe 1 all Δb* are also negative. For Probe 1, the Δa* are positive, which makes sense because Probe 1 corresponds to a green color, so in this case higher a* values after the cleaning implies also less saturated green color after the cleaning. Finally, we also see that L* is always higher, in agreement with the conclusions derived from the analysis of the sample’s spectral reflectance curves.

### 3.3. Pixelwise Spectral Reflectance and L*, a*, b* Values

**Figure 6:**
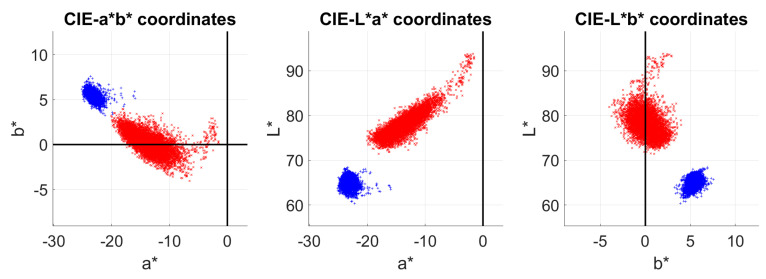
2D projections of the L*, a*, b* color space plotting pixel-wise color information of sample 1 in probe 1, before (blue) and after (red) cleaning.

In addition to the color difference from mean L*, a*, b* values, the spectral imaging system used for this research is able to deliver spectral reflectance curves for each pixel of the image. Thus, by computing the corresponding L*, a*, b* values for each reflectance in the retrieved 100 × 100 pixels area, we achieve 10,000 color points that make up part of a “color cloud”. This is shown in Figure 6 where the color clouds of sample 1 in probe 1 are shown before (blue) and after (red) the cleaning.

**Figure 7:**
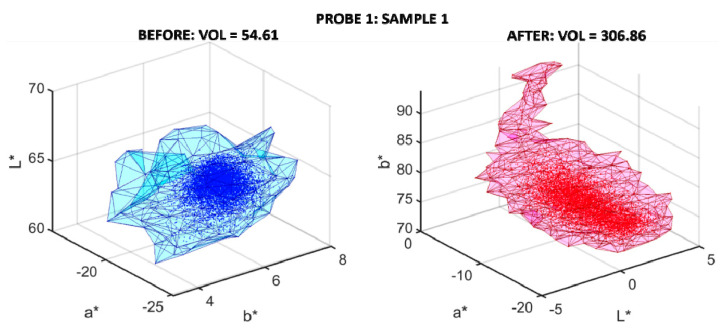
Minimum volume of the L*, a*, b* clouds of sample 1 of probe 1 before (**left**) and after (**right**) cleaning.

We could consider the center of mass of each cloud in Figure 6 as the mean color for these samples. It is evident how the point clouds become more spread across the color space after the cleaning. This result cannot be found by analyzing the mean color difference results, as we did in the previous section. The increase in cloud size reflects that the cleaning is leaving some white residue on the sample, and that the spatial distribution of this residue is not very homogeneous, so that some points are less white than others. This spread or heterogeneity can be measured by calculating the standard deviation of the L*, a*, b* color coordinates as well as calculating the minimum volume of the point clouds. Figure 7 shows an example of the minimum volume [38,39] calculated for sample 1 in probe 1, and Table 4 contains the mean standard deviation (std) across the wavelengths and the mean standard deviation of the L*, a*, b* color coordinate clouds, as well as the minimum volumes of the point clouds. Higher values of std or volume indicate higher heterogeneity of the samples after the cleaning process. Note that this information is complementary to that shown in Figure 5, where the standard deviation is plotted wavelength-wise to see what wavelengths are affected more or less by the heterogeneity introduced in the cleaning processes.

As shown in Table 4, for probe 1, cleaning process number 3 (AB-57 + scalpel) produces the highest heterogeneity, and in general number 7 (complex polysaccharide gel (Agar-Agar^®^) + scalpel) the lowest. For probe 2, cleaning process number 9 (glass fiber pencil) in general produces the highest heterogeneity, but the lowest heterogeneity is found with method 4 (polyacrylic acid gel + scalpel). The results for the minimum volume increment ratio are different from the results based on L*a*b* std values, but the methods of choice according to L*a*b* std (indicated above) are also among the three best or three worst according to minimum volume increment.

### 3.4. Amount of White Residue Left after the Cleaning

**Figure 8:**
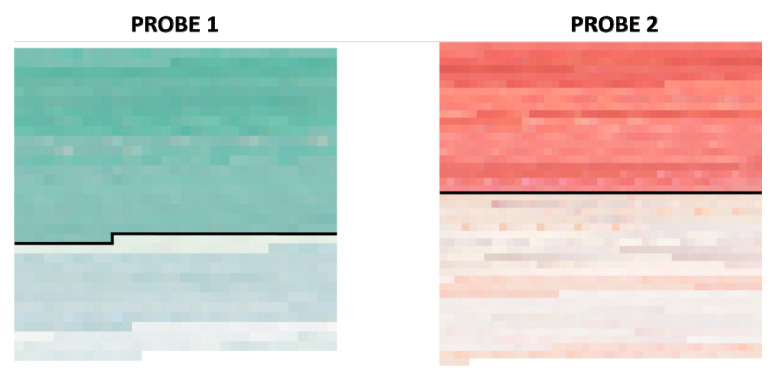
sRGB renderization of the manually retrieved ground truth set of pigments (above black line) and the white (below black line) pixels from both probes.

For the validation of the k-means classification, white and pigment areas were manually extracted and pooled together for each probe in order to perform the same k-means algorithm. Figure 8 shows the sRGB renderization of the ground truth sets of pixels retrieved from different samples in both probes. The pigment (top) and white (bottom) pixels are separated by a black line for each set. 1036 samples were extracted from probe 1:637 green (top) and 399 white (bottom), and 1810 samples from probe 2:860 red (top) and 950 white (bottom).

**Figure 9:**
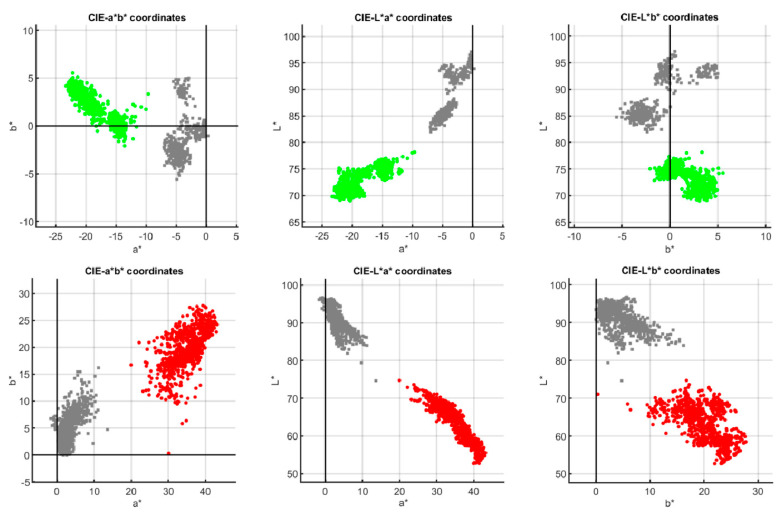
L*, a*, b* clouds for samples retrieved from probe 1 (top row) and probe 2 (bottom row). The green and red colors represent pigment pixels and the grey color represents the white pixels.

Figure 9 shows the L*, a*, b* clouds of both sets of pixels (pigment and white) for both probes. The top row shows the three two-dimensional projections of such a color space for probe 1. The green color represents pigment samples and the grey color the white samples. The bottom row shows the same for probe 2 (the red color pigment samples and the grey color for the white samples). As can be observed, the different samples are easily classifiable with the k-means algorithm.

The k-means algorithm was able to correctly classify 100% of the pixels from both ground truth sets as either pigment or white pixels. This result highlights the confidence in the information shown in Table 5, as a figure of merit for the different cleaning methods used for both probes.

**Figure 10:**
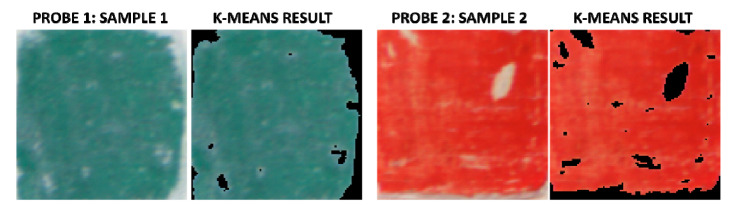
sRGB renderization of cleaned areas and the same with highlighted automatically segmented white areas (black color) of sample 9 in probe 1, and sample 2 in probe 2.

Regarding the results of the automatic segmentation of the white areas, Figure 10 shows example results of two different samples (sample 3 in probe 1 and sample 2 in probe 2). In this figure, automatically segmented white areas are highlighted in black (for easier visualization). In general, the results are satisfactory for all the samples and most white areas visually detectable were correctly segmented.

Table 5 shows the percentage of automatically segmented white areas in all samples of both probes. Since these white areas are considered areas where the cleaning process failed, the higher this percentage is the worse the cleaning process performed.

As Table 5 shows, cleaning method number 9 (glass fiber pencil), followed by method number 3 (AB-57 + scalpel) performs best for probe 1. Method number 6 (cotton swab + scalpel) performs worst for probe 1, producing around seven times more white pixels. For probe 2, method number 1 (scalpel) offers the best result, followed by method number 4 (polyacrylic acid + scalpel), whilst the worst method is number 9 (glass fiber pencil). Adding up the percentage of both probes, the cleaning method that produced the least white areas was method number 8 (Hydroxypropyl cellulose gel + scalpel). The worst cleaning method according to this figure of merit and taking into account the two probes is method number 6 (cotton swab + scalpel).

## 4. Discussion

The results offered by the standard deviation analysis, both in spectral and L*, a*, b* data (see Section 3.1 and Section 3.3), are quite in agreement: for the malachite probe (1), the best performing method (less inhomogeneity) is number 7 (complex polysaccharide gel (Agar-Agar^®^) + scalpel), whilst the methods that produce more inhomogeneity are number 9 (glass fiber pencil) and 3 (AB-57 + scalpel). For the cinnabar sample, the method of choice (most homogeneous results across sample pixels) would be number 4 (polyacrylic acid gel + scalpel) followed by 1 or 2 (both cellulose based), whilst the least homogeneous results would be those obtained with method 9 (glass fiber pencil).

The minimum volume increment analysis and the residual white analysis show that the best performing method would be number 9 (glass fiber pencil) for the malachite samples, whilst the method of choice would be number 1 (methylcellulose gel + scalpel) for the cinnabar samples. The worst methods are number 3 (AB-57 + scalpel) and 6 (cotton swab + scalpel) for the malachite sample, whilst number 6 (cotton swab + scalpel) and number 9 (glass fiber pencil) are the worst for the cinnabar sample.

According to the color difference analysis, however, the best performing method for the malachite sample would be number 3 (AB-57 + scalpel), and the worst method, number 6 (cotton swab + scalpel); for the cinnabar sample, the methods of choice would be numbers 1(Methylcellulose gel +scalpel), 2(Cellulose poultice + scalpel) or 7 (Complex polysaccharide gel (Agar-Agar^®^) + scalpel), whilst the worst performing would be number 9 (glass fiber pencil).

The discrepancies found can be explained if we consider that the different analysis is pinpointing different aspects (points in favor or against a given method). For instance, for the malachite sample, we can see how method number 3 (AB57 + scalpel) would never be chosen if we look at the inhomogeneity results; however, it would be considered if we wished for a more similar mean color to the original sample after the cleaning process. If we look at the sRGB renderization of the samples both before and after the cleaning process (see Figure 4), we can see how the more similar color to the original sample is indeed method number 3 (AB57 + scalpel); nevertheless, with this method, some clear patches of white residue have been left after the cleaning, and hence the high values of inhomogeneity indexes would not be an asset of this method. This conclusion about method 3 for Probe 1 is supported as well by the spectral metrics pixel-by-pixel analysis: looking at the average results of GFC and RMSE, most of the pixels have a spectrum which is similar to the original probe color, and thus the average GFC and RMSE are better for this method. When the performance of a method is clearly below par, however, all the analyses agree in ranking it among the worst performing: this is what happened with method number 9 (glass fiber pencil) and the cinnabar sample. For this same sample, taking into account the results produced by all the methods tested, the best performing method would be number 1 (methylcellulose gel +scalpel) followed by number 4 (Polyacrylic acid gel + scalpel).

As mentioned above, the identification of a layer of color which was less consistent in Probe 2 has notably influenced the results as it was more sensitive to the processes involving a chemical agent which softens the surface or a strong abrasive action such a glass fiber pencil. The results confirm that mechanical cleaning using a scalpel is a valid method for these surfaces if there is good consistency of the original polychromy layer to be cleaned on the plasterwork. This action can be improved with the use of a gel formed from the complex polysaccharide Agar-Agar^®^, as the scalpel alone can be a very aggressive treatment. In general, in both cases the glass fiber pencil is the least recommended method.

## 5. Conclusions

Using a non-invasive spectral imaging capture device has produced a much more comprehensive analysis than conventional spectrophotometry on the efficiency of different cleaning methods to eliminate a white layer deposited on a pigment layer on plaster probes.

Due to the many analytical methods tested, this study is restricted to two pigments that were commonly used in the Nasrid period (malachite and cinnabar) and the two binders most commonly used (animal glue and gum Arabic). It would be of interest to extend the study to additional pigments and the same binders and also to consider additional analytical methods that would characterize the spatial inhomogeneity of the sample, such as texture-based analysis.

We have introduced four different analytical methods: standard deviation across wavelengths, L*, a*, b* standard deviation, minimum volume of the color clouds and amount of white residue left. Moreover, we have also computed the CIEDE2000 color difference both before and after and the cleaning processes, as well as the differences in L*, a* and b*, using the center of mass of the color clouds.

The data extracted from the conducted comparisons confirm the difficulty of analyzing what are the most effective processes on works of art. This is due primarily to the diversity of the materials and the different response they have over time. A first visual analysis after applying the treatments (Figure 4) allows us to state that the malachite color layer of Probe 1 is much more adhered than the cinnabar layer applied on Probe 2. In this case, in addition to the influence the composition of the pigment might have, the difference in adherence is due mainly to the binder used, as this is the element which makes it adhere to the surface. Animal glue (Probe 1) is also a more stable and resistant binder than gum Arabic (Probe 2), both initially and as time goes by. For this reason, it would be important to analyze and consider the composition of the material and the consistency of the color layers previously with the aim of selecting the most adequate treatments with regards to the materials.

This paper highlights the necessity to continue studying the effects of cleaning processes for different problems and the importance this information has for restorers before starting restoration intervention on works of art with these characteristics.

## Figures and Tables

**Table 1:** Selected treatments for each probe.

PROBE 1				PROBE 2			
#	Method	Solvent	Time (min)	#	Method	Solvent	Time (min)
**1**	Scalpel	x	X	**1**	Methylcellulose gel + scalpel	Water and acetone (75:25)	30
**2**	Cellulose poultice + scalpel	Distilled water	20	**2**	Cellulose poultice + scalpel	Water and acetone (75:25)	30
**3**	AB-57 + scalpel	x	20	**3**	AB-57 + scalpel	x	30
**4**	Polyacrylic acid gel + scalpel	Distilled water	20	**4**	Polyacrylic acid gel + scalpel	Water and acetone (75:25)	30
**5**	Gel from complex polysaccharide (Gellano Kelogel^®^) + scalpel	Distilled water	20	**5**	Gel from complex polysaccharide (Gellano Kelogel^®^) + scalpel	Water and acetone (75:25)	30
**6**	Cotton swab + scalpel	Distilled water	X	**6**	Cotton swab + scalpel	Water and acetone (75:25)	X
**7**	Complex polysaccharide gel (Agar-Agar^®^) + scalpel	Distilled water	20	**7**	Complex polysaccharide gel (Agar-Agar^®^) + scalpel	Water and acetone (75:25)	30
**8**	Hydroxypropyl cellulose Gel +scalpel	Distilled water	20	**8**	Hydroxypropyl cellulose gel + scalpel	Water and acetone (75:25)	30
**9**	Glass fiber pencil	x	X	**9**	Glass fiber pencil	x	X

**Table 2:** Mean ΔE00 color difference for each sample of both probes: before cleaning vs. after cleaning.

Sample	1	2	3	4	5	6	7	8	9	Mean
Probe 1	12.0	10.2	8.0	12.6	10.6	16.9	11.2	11.5	12.5	11.7
Probe 2	11.8	11.8	19.9	12.4	16.9	12.1	11.8	14.0	25.3	15.1

**Table 3:** Differences in average color coordinates L*, a* and b* for the 2 probes and the 9 cleaning methods.

**Probe 1**									
**Method**	**1**	**2**	**3**	**4**	**5**	**6**	**7**	**8**	**9**
ΔL*	12.9	10.4	9.3	13.7	11.0	16.1	11.3	10.6	12.3
Δa*	9.6	9.6	5.4	10.1	9.7	15.8	9.9	11.6	11.6
Δb*	−5.2	−4.3	−2.8	−5.2	−4.8	−7.1	−5.7	−5.8	−5.6
**Probe 2**									
**Method**	**1**	**2**	**3**	**4**	**5**	**6**	**7**	**8**	**9**
ΔL*	12.5	12.7	21.9	13.5	18.4	13.2	12.8	15.4	27.7
Δa*	−8.1	−7.8	−16.1	−8.4	−15.0	−6.0	−6.8	−10.7	−23.7
Δb*	−5.8	−3.0	−6.2	−5.3	−8.5	−2.6	−3.6	−5.4	−12.7

**Table 4:** Standard deviation of the spectral reflectances (mean across all wavelengths) and the L*, a*, b* color coordinates, and L*, a*, b* clouds volumes before and after cleaning for all the samples.

**PROBE 1**										
**Sample**	**std Ref**		std L*		std a*		std b*		**Volume Lab**	
	**Before**	**After**	**Before**	**After**	**Before**	**After**	**Before**	**After**	**Before**	**After/Before**
1	0.010	0.05	0.9	2.6	0.6	2.6	0.4	1.1	54.6	5.6
2	0.008	0.05	0.7	2.9	0.6	3.1	0.4	1.4	46.0	20.0
3	0.010	0.07	1.0	**4.3**	0.6	**3.6**	0.6	**2.0**	65.3	**30.2**
4	0.012	0.06	1.3	3.0	0.6	3.0	0.6	1.2	38.3	13.6
5	0.009	0.05	0.8	2.4	0.6	2.6	0.4	1.1	41.4	25.1
6	0.009	0.08	0.8	3.6	0.7	**3.6**	0.6	1.8	105.8	8.6
7	0.014	**0.04**	1.4	**2.4**	0.7	**2.0**	0.6	**0.9**	86.2	6.5
8	0.012	0.06	1.1	3.0	0.7	2.9	0.5	1.1	89.3	6.5
9	0.012	0.09	1.2	4.2	0.7	3.2	0.8	1.2	141.9	**3.7**
**PROBE 2**										
**Sample**	**std ref**		std L*		std a*		std b*		**Volume Lab**	
	**Before**	**After**	**Before**	**After**	**Before**	**After**	**Before**	**After**	**Before**	**After/Before**
1	0.016	0.10	1.6	8.0	1.5	8.1	1.5	**4.1**	595.0	**5.7**
2	0.013	**0.08**	1.2	7.4	1.5	8.9	1.4	4.3	340.0	12.0
3	0.013	0.09	1.4	7.2	1.2	9.2	1.3	4.7	184.9	31.6
4	0.013	**0.08**	1.3	**6.7**	1.2	**7.9**	1.3	**4.1**	569.1	7.8
5	0.014	0.11	1.5	8.3	1.2	**9.7**	1.1	4.5	264.2	12.1
6	0.016	0.09	1.7	7.5	1.4	8.7	1.5	4.5	251.4	**32.4**
7	0.016	0.10	1.6	7.9	1.7	9.1	1.9	4.8	759.7	8.2
8	0.017	0.09	1.6	7.7	2.0	9.1	1.8	4.7	531.5	5.9
9	0.013	**0.12**	1.4	**8.5**	1.2	9.3	1.4	**5.1**	226.2	18.4

**Table 5:** Percentage of automatically segmented white areas over the full area studied for all the samples and probes. The higher this value is the worse the performance of the cleaning method. The bottom row shows the total of the two previous rows.

Sample	1	2	3	4	5	6	7	8	9
Probe 1	45.27	44.41	15.32	26.06	32.36	**63.14**	28.02	16.66	**9.30**
Probe 2	**6.81**	11.42	32.86	7.87	17.12	14.20	9.21	10.50	**41.85**
Total	52.08	55.83	48.18	33.93	49.48	77.34	37.23	**27.16**	51.15
